# Antimicrobial resistance trends of methicillin-resistant *Staphylococcus aureus* in Norway from 2008 to 2017

**DOI:** 10.1093/jacamr/dlaf094

**Published:** 2025-06-06

**Authors:** Torunn Gresdal Rønning, Hege Enger, Jan Egil Afset, Christina Gabrielsen Ås

**Affiliations:** The Norwegian MRSA Reference Laboratory, Department of Medical Microbiology, Clinic of Laboratory Medicine, St. Olavs Hospital, Trondheim University Hospital, Trondheim, Norway; Department of Clinical and Molecular Medicine, Norwegian University of Science and Technology, Trondheim, Norway; Department of Medical Microbiology, Clinic of Laboratory Medicine, St. Olavs Hospital, Trondheim University Hospital, Trondheim, Norway; The Norwegian MRSA Reference Laboratory, Department of Medical Microbiology, Clinic of Laboratory Medicine, St. Olavs Hospital, Trondheim University Hospital, Trondheim, Norway; Department of Medical Microbiology, Clinic of Laboratory Medicine, St. Olavs Hospital, Trondheim University Hospital, Trondheim, Norway; Department of Clinical and Molecular Medicine, Norwegian University of Science and Technology, Trondheim, Norway; Department of Medical Microbiology, Clinic of Laboratory Medicine, St. Olavs Hospital, Trondheim University Hospital, Trondheim, Norway; The Norwegian MRSA Reference Laboratory, Department of Medical Microbiology, Clinic of Laboratory Medicine, St. Olavs Hospital, Trondheim University Hospital, Trondheim, Norway; Department of Clinical and Molecular Medicine, Norwegian University of Science and Technology, Trondheim, Norway; Department of Medical Microbiology, Clinic of Laboratory Medicine, St. Olavs Hospital, Trondheim University Hospital, Trondheim, Norway

## Abstract

**Objectives:**

The purpose of this study was to analyse nationwide trends in antimicrobial susceptibility and molecular epidemiology of all MRSA strains in Norway over a 10-year period.

**Materials and methods:**

All cases of MRSA in Norway from 2008 to 2017 were included, limited to the first case per year per individual (*n* = 15 200). Strains were confirmed as MRSA with PCR and genotyped using *spa*-typing. Antimicrobial susceptibility data and epidemiological data were collected from the Norwegian MRSA reference laboratory and the Norwegian Surveillance System for Communicable Diseases, respectively.

**Results:**

Despite an increase in MRSA cases, antimicrobial resistance rates remained stable throughout the study period. The most common susceptibility profile of the MRSA strains was resistance to cefoxitin alone (41.4%), while co-resistance (58.6%) was observed most commonly for erythromycin (31.0%), tetracycline (25.3%) and ciprofloxacin/norfloxacin (21.6%). Notably, associations were identified between specific resistance patterns and clinical variables, including higher resistance rates among healthcare-associated MRSA, particularly in older adults and nursing home residents. Geographic associations were also observed, linking specific resistance profiles to strains acquired in North America, Africa and Asia.

**Conclusions:**

The findings highlight a complex landscape of MRSA resistance in a low-prevalence country, characterized by a diverse genotypic population and stable longitudinal trends in resistance rates. However, the prevalence of co-resistance to one or more antibiotics among Norwegian MRSA strains was high and increasing. This study thus underscores the importance of continuous surveillance and adherence to antimicrobial guidelines.

## Introduction

*Staphylococcus aureus* is part of the normal human bacterial flora, with carriage rates of 20%–30%, predominantly within the nose and throat and on skin. *S. aureus* is, however, also recognized as a significant human pathogen, associated with a wide variety of infections ranging from mild skin and soft-tissue conditions to severe systemic disease.^1^ The pathogen’s extensive repertoire of virulence factors is key in facilitating colonization, invasion and evasion of host immune defences. Furthermore, *S. aureus* exhibits remarkable adaptability to diverse environments and host organisms. Combined with its propensity to rapidly develop resistance to commonly used antimicrobial agents, these factors have led to its position as being the leading bacterial cause of death globally.^2^

Historically, there have been several important events of antimicrobial resistance (AMR) development in *S. aureus.*^3^ Shortly after the seminal introduction of penicillin as antimicrobial treatment in the early 1940s, strains of penicillin-resistant *S. aureus* emerged, initially within hospital settings and subsequently in the community. Similarly, after the penicillinase-stable antibiotic methicillin was introduced in 1961, methicillin-resistant *S. aureus* (MRSA) infections were detected within a year. Since then, multiple MRSA clones have emerged and spread globally. Today, MRSA continues to pose a significant public health challenge across high- and low-income countries, characterized by limited treatment options, elevated mortality and morbidity^2^ and a marked potential for transmission and causing outbreaks.^1^

Norway is a low-prevalence country in terms of MRSA, exhibiting a highly heterogenous MRSA population.^4^ The reporting of MRSA infections and colonization has been mandatory since 1995 and 2005, respectively. Since 2008, all MRSA strains identified in Norway have been collected, confirmed, and genotyped at the Norwegian MRSA reference laboratory. Together, this systematic data collection offers valuable insights into national longitudinal trends in MRSA epidemiology and antimicrobial resistance. With this study, we thus aim to contribute to national and global surveillance of MRSA, which is important for informing treatment strategies as welld as guidelines and infection control measures aimed at mitigating the dissemination of MRSA, both within healthcare settings and in the community.

## Materials and methods

### Ethics

The project was approved with exemption from informed consent of participants (reference number 205 233), by the Norwegian Regional Committees for Medical and Health Research Ethics (REC) Middle Norway. A Data Protection Impact Assessment was performed and approved by the responsible institution, the Clinic of Laboratory Medicine at St. Olavs Hospital, Trondheim University Hospital.

### Study design and population

A case of MRSA was defined as laboratory-confirmed MRSA either through notification to the Norwegian Surveillance System for Communicable Diseases (MSIS) and/or confirmation by the Norwegian MRSA reference laboratory. All cases of MRSA in Norway from 2008 to 2017 were included, but limited to the first case per year per individual.

### Clinical and epidemiological data

Clinical and epidemiological data on all cases were collected from MSIS and the request forms from the referring laboratory or the treating physician (accessed from the Norwegian MRSA reference laboratory). The information from MSIS included age, sex, admission to hospital or nursing home, assumed place of acquisition of MRSA and whether part of a known outbreak. The data obtained from the Norwegian MRSA reference laboratory included sample date, sampling site/type of sample and laboratory results. The MRSA cases were categorized as carriage, infection, invasive infection or unknown based on sampling site/type of sample. Age groups were based on categories defined by Diaz *et al.*^5^ Due to a lack of data on admission times of hospitalized and nursing home patients, a broad definition of health care-associated MRSA (HA-MRSA) was used. Accordingly, a case was categorized as HA-MRSA if MRSA was detected during a hospital or nursing home stay, or in healthcare workers, while community-acquired MRSA (CA-MRSA) was defined as all remaining cases. The clinical and epidemiological dataset was previously investigated in a separate study.^4^

### *Bacterial strains, PCR and* spa*-typing*

Bacterial strains were cultured on blood agar at 35°C, after which extraction of DNA was performed by heat lysis. A few colonies were suspended in molecular grade water and heated to 95°C for 15 minutes with shaking (300 rpm). After centrifugation at 14 500 rpm for 2 minutes, the supernatant was collected. Confirmation of MRSA was performed with a multiplex conventional PCR detecting the *mecA* gene, the *S. aureus*-specific *spa* gene and the Panton–Valentine leukocidin genes *luk*SF-PV, followed by gel electrophoresis.^6^ For strains that were *mecA* negative, *mecC* PCR was additionally performed.^7^ All strains were *spa*-typed according to Harmsen *et al.*^6^ with primers *spa*-1113f and *spa*-1514r^8^ using the Ridom StaphType software and SpaServer.^9^ The *spa*-types were assigned to known sequence types (ST) and/or clonal complexes (CC) based on Ridom Staphtyper^8^ and pubMLST^10,11^ databases. If a *spa*-type could not be assigned to a ST or CC, multi-locus sequence typing was performed, and CC was assigned using eBURST software.^12^ The genotypic dataset was previously characterized in a separate study.^4^

### Antimicrobial susceptibility testing

Antimicrobial susceptibility testing (AST) was performed at the Norwegian MRSA reference laboratory (2009–2015) or the referring laboratory (2016–2017) using the European Committee on Antimicrobial Susceptibility Testing (EUCAST) disc diffusion method^13^ or agar gradient method,^14^ for all strains. Inducible clindamycin resistance (ICR) was registered after 2011.^15^ Results were interpreted as either susceptible, intermediate or resistant according to the 2017 EUCAST break point table v.7.0. The following antibiotic discs (Oxoid) were included in the panel: cefoxitin, erythromycin, clindamycin, fusidic acid, gentamicin, tetracycline, trimethoprim/sulfamethoxazole, ciprofloxacin/norfloxacin, rifampicin, mupirocin and linezolid. The D-zone reaction for testing of ICR was also recorded as described in the EUCAST expert rules.^16^ For vancomycin, the gradient strip test from BioMeriéux (2008–2013) or Liofilchem (2014–2017) was used, according to the manufacturer’s instructions. *S. aureus* CCUG 15 915 was used as control strain to monitor AST performance. The term co-resistance was used as per the definition of the WHO Advisory Group, ‘resistance to more than one antimicrobial agent of the same or different classes’.^17^

### Statistics

Fisher’s exact test was used for testing associations between antibiotic resistance and epidemiological variables (infection/carriage, HA/CA, sex and country/continent of acquisition). Binary logistic regression and the Chi-Square test (cross-table analysis) was used for testing interactions between fluoroquinolone resistance, age group and sex. Statistical analyses were performed using R studio v.1.4 and IBM SPSS Statistics v.29.0.0.0. The Benjamini–Hochberg method was used to correct for multiple hypothesis testing, with adjusted *P* values <0.05 regarded as statistically significant.

## Results

### Antimicrobial resistance rates and longitudinal trends

In total, there were 15 200 cases of MRSA from 14 386 people in the study period from 2008 to 2017.^4^ Of the MRSA strains, 12 128 (79.8%) had AST data (Figure S1, available as Supplementary data at *JAC-AMR* Online), combined with near complete *spa*-typing data covering 99.99% of these strains. Antimicrobial susceptibility data, were missing mainly from the earliest years in the period (2008, 2010 and 2011), where <5% of cases were tested. For 2009, results were available from 75.8% of cases, and for the remaining years of the study period >95.0% of cases were tested per year.

All strains were PCR-positive for either the *mecA-* (12 112 strains, 99.9%) or the *mecC* gene (16 strains, 0.1%).^4^ All strains were phenotypically resistant to cefoxitin (99.9%), except seven susceptible strains constituting oxacillin sensitive MRSA. The cefoxitin susceptible strains (0.06%) displayed AST inhibition zones close to the breakpoint of cefoxitin (22–24 mm), and represented different *spa*-types and collection years. Such strains should, however, be reported as cefoxitin resistant, given that they are probably capable of reverting to a resistant phenotype on exposure to cefoxitin.^18,19^

**Figure 1.**
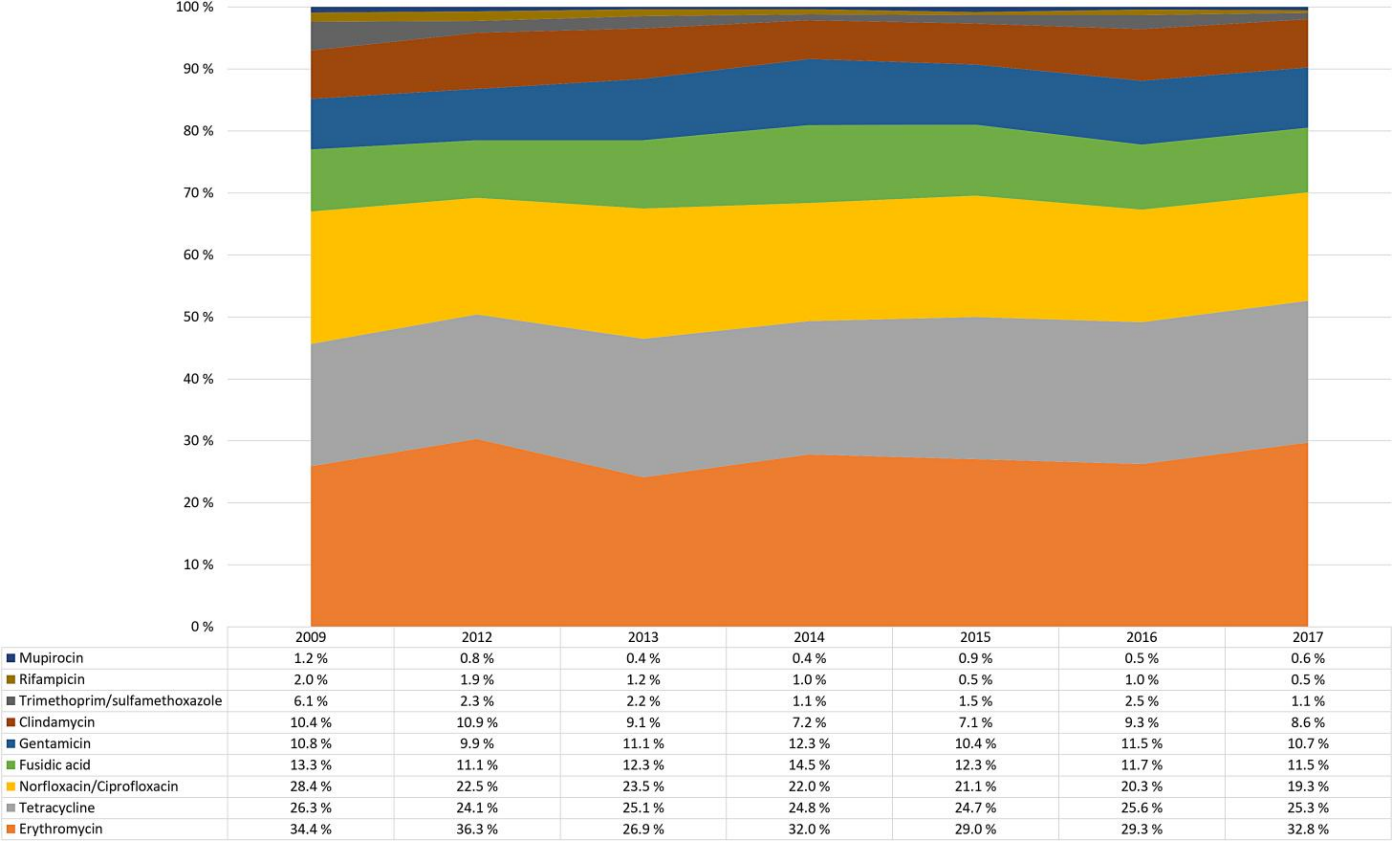
Yearly resistance rates of Norwegian MRSA strains shown for selected antibiotics during the period 2009–2017. ICR was first registered after 2011, and is therefore not part of the data from 2009.

Despite a major increase in the number of MRSA cases in the study period, the overall antimicrobial resistance rates to the panel of antibiotics tested were stable, with no major changes over time (Figure 1). The highest co-resistance rates were observed for erythromycin (31.0%), tetracycline (25.3%), ciprofloxacin/norfloxacin (21.6%) and clindamycin (21.3%). Total clindamycin resistance included constitutive resistant (8.6%) and ICR strains (12.7%). Moderate resistance levels were observed for fusidic acid and gentamicin, with 12.4% and 11.0% resistance, respectively. Low rates of resistance were observed for trimethoprim/sulfamethoxazole (2.0%), rifampicin (1.0%), mupirocin (0.6%) and linezolid (0.008%). No reduced susceptibility to vancomycin was detected.

### Co-resistance and AMR profiles of MRSA genotypes

**Figure 2.**
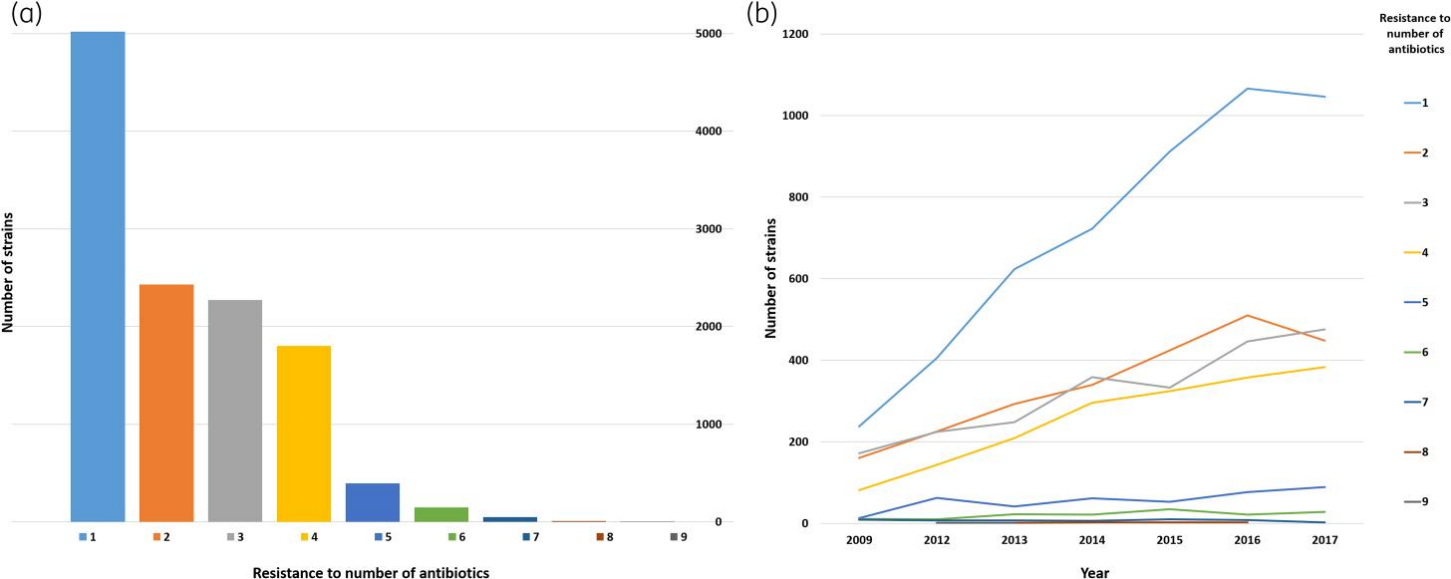
(a) Number of MRSA strains and (b) strains per year that are phenotypically resistant against cefoxitin alone (1) or multiple antibiotics, as indicated by the colour key.

**Figure 3.**
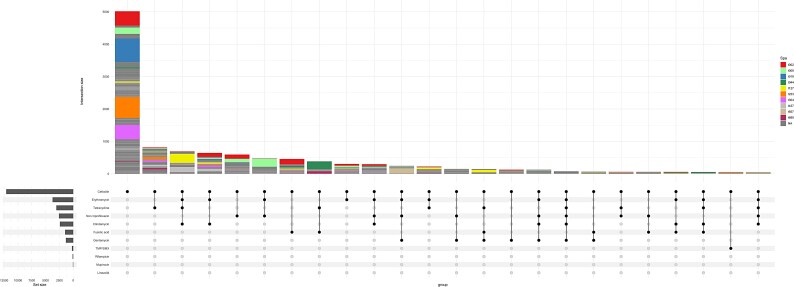
Upset plot showing the most common combinations (>50 cases) of AMR phenotypes in MRSA strains. The 10 major *spa*-types are coloured according to the key.

The most common susceptibility profile of the MRSA strains was resistance to cefoxitin alone (*n* = 5021, 41.4%) (Figures 2a and 3), and the yearly number of such strains appeared to increase throughout the study period (Figure 2b). However, co-resistance to one or more additional antibiotics was common, with 38.8% of strains (*n* = 4703) displaying resistance to two or three antibiotics, and 14.9% of strains (*n* = 1802) resistant to four antibiotics. These strains appeared to increase moderately in the study period (Figure 2b). Only 5.0% of strains (*n* = 602) were resistant to five or more antibiotics, and the occurrence of such strains was stable throughout the study period. The most broadly co-resistant strains (*n* = 3) were resistant towards nine antibiotics, including cefoxitin, erythromycin, clindamycin, gentamicin, ciprofloxacin/norfloxacin, fusidic acid, tetracycline and trimethoprim-sulfamethoxazole as well as rifampicin.

The most common phenotypic AMR profiles, aside from cefoxitin resistance alone, were co-resistance to tetracycline (6.7%), followed by co-resistance to erythromycin, clindamycin and tetracycline, accounting for 5.7% of cases (Figure 3). Co-resistance to erythromycin and clindamycin (5.3%) was also frequent, while co-resistance to erythromycin alone was noted only in 2.5% of cases. Last, co-resistance to ciprofloxacin/norfloxacin alone (4.9%) or in combination with erythromycin (3.9%) were among the prevalent AMR profiles.

**Figure 4.**
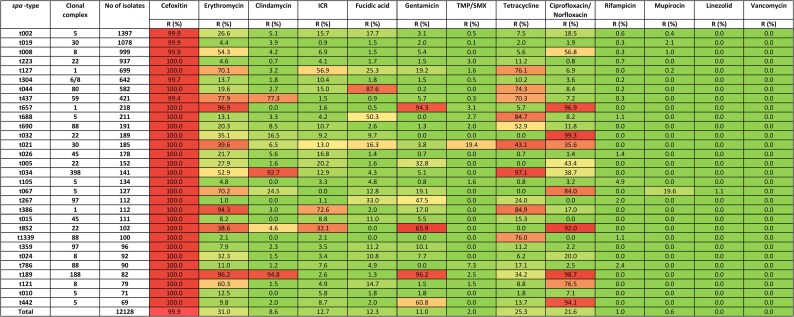
Resistance rates to the tested antibiotics among the 30 most prevalent MRSA *spa*-types and in all strains during the period 2008–2017. The number of strains in column three represents the number of strains tested for cefoxitin. TMP/SMX, trimethoprim/sulfamethoxazole.

There was considerable heterogeneity of MRSA genotypes detected in the study period.^4^ However, the AMR profiles of some of the most common *spa*-types appeared to be stable and predictable (Figure 4). Of these, MRSA t002, t019, t223 and t304 had low resistance rates to antibiotics other than cefoxitin, while MRSA t008, t127, t044, t437, t657 and t688 had high rates of co-resistance to 2–3 antibiotics. Among the most common genotypes, MRSA t189 had high co-resistance rates against four antibiotics, including erythromycin, clindamycin, gentamicin and ciprofloxacin/norfloxacin.

### Genotypes contributing to AMR for each antibiotic group

**Figure 5.**
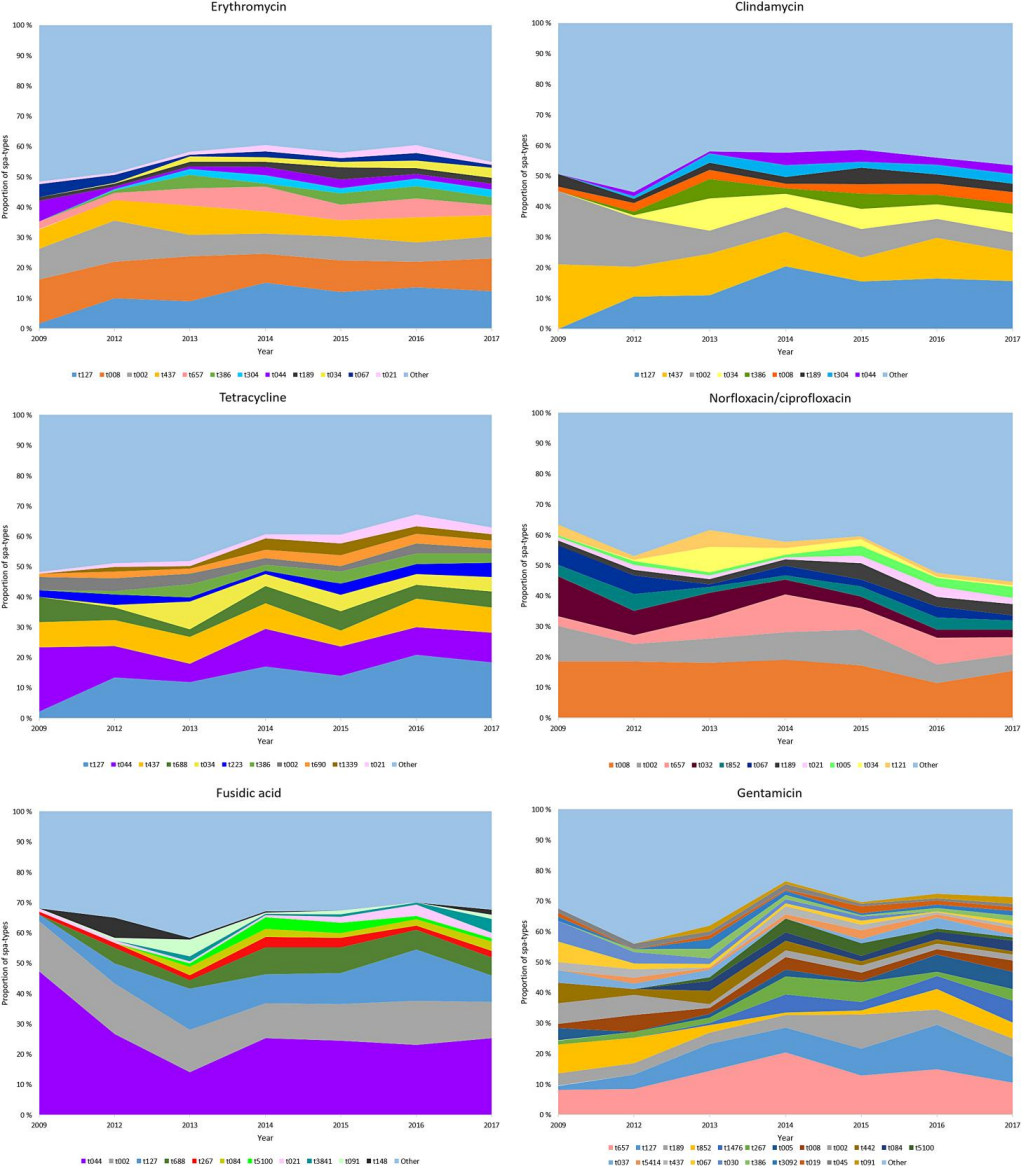
Yearly distribution of MSRA genotypes contributing to resistance to selected antibiotics. Genotypes found <50 times per year are designated as ‘other’ for erythromycin, clindamycin, norfloxacin/ciprofloxacin and tetracycline, while genotypes found <15 times per year were designated as ‘other’ for fusidic acid and gentamicin.

Similar to the observed heterogeneity of MRSA *spa*-types in the study period, for most of the tested antibiotics, there were multiple genotypes contributing to resistance and no single clones that dominated (Figures 3 and 5). We found no major temporal shifts in genetic epidemiology that had any pronounced effect on AMR during the study period, except a decrease in fusidic acid resistant MRSA t044 in the beginning of the period (2013) (Figure 5). Of all erythromycin-resistant MRSA strains, t127, t008 and t002 were the major *spa*-types, while for norfloxacin/ciprofloxacin resistance, t008, t002 and t657 contributed most. For clindamycin resistant strains, generally the same pattern of resistance was seen as for erythromycin: t127, t437 and t002 were the most resistant strains. An exception was the livestock associated t034, which displayed relatively more resistance to clindamycin than to erythromycin (Figures 4 and 5). Of tetracycline-resistant MRSA strains t127, t044 and t437 were most frequent, and for fusidic acid resistance, t044, t002 and t127 were the dominant *spa*-types. For gentamicin resistant strains, t657 and t127 contributed the most.

### Associations between AMR and epidemiology

Based on statistical analyses of clinical and epidemiological variables, we observed significant associations between erythromycin resistance and infection (Table 1). Resistance to fluoroquinolones was furthermore significantly associated with HA-MRSA (Table 1, Figure S2), predominantly with people admitted to nursing homes (data not shown). Resistance to fluoroquinolones was also significantly associated with age group (*P* < 0.001), specifically for the age group ‘older adults’ (≥60 years) (*P* < 0.001) (Figure S3). Moreover, statistical analyses of place of acquisition (Table 1, Figure S4) highlighted that MRSA strains exhibiting co-resistance to erythromycin were highly significantly associated with North America. Similarly, co-resistance to fusidic acid and tetracycline was significantly linked to strains from the African continent, and gentamicin resistance was significantly associated with strains from Asia (Table 1).

**Table 1.** Association between resistance to different antibiotics and clinical and epidemiological data for MRSA cases in Norway in 2008–2017

Clinical or epidemiological variable	Antibiotic	*n* ^a^	*P* value^b^
Healthcare-associated	Ciprofloxacin/norfloxacin resistance	927	2.09E-17
	Resistance against 5 or more antibiotics	206	1.00E-02
Infection	Erythromycin resistance	1490	1.52E-13
	Ciprofloxacin/norfloxacin resistance	975	1.71E-04
Female	Ciprofloxacin/norfloxacin resistance	1221	3.00E-02
Africa	Tetracycline resistance	142	2.07E-13
	Fusidic acid resistance	85	3.27E-12
	Gentamicin resistance	66	1.11E-06
	Ciprofloxacin/norfloxacin resistance	50	5.89E-04
	Erythromycin resistance	76	1.37E-03
Asia	Gentamicin resistance	244	2.04E-11
	Resistance against 5 or more antibiotics	122	2.24E-08
	Fusidic acid resistance	137	2.96E-05
	Ciprofloxacin/norfloxacin resistance	306	1.89E-04
	Tetracycline resistance	339	5.73E-03
North America	Erythromycin resistance	60	4.78E-17
South- and Middle-America	Erythromycin resistance	79	1.34E-07
Europe (excluding Norway)	Clindamycin total resistance	216	1.20E-07
	Ciprofloxacin/norfloxacin resistance	224	1.20E-07
	Erythromycin resistance	291	2.82E-04
	Gentamicin resistance	70	7.61E-04
Age group (all)	Ciprofloxacin/norfloxacin resistance	2593	<0.001
Age group older adults	Ciprofloxacin/norfloxacin resistance	743	<0.001

^a^ *n* < 50 are not included.

^b^Benjamini–Hochberg-corrected *P* <0.05 regarded as significant

## Discussion

Norway has a low incidence of MRSA with 48.6 cases per 100 000 person years in 2017.^4^ Although the number of MRSA cases has increased significantly in the study period, the relative resistance rates of the tested antibiotics were stable throughout the period. This is in contrast to findings from a Swiss study with similar sample size, where rates of resistance to ciprofloxacin, clindamycin, gentamicin and erythromycin were found to be decreasing in an overlapping time period.^20^ Many of the major genotypes in our study displayed predictable antibiotic resistance profiles, however, the large heterogeneity of MRSA genotypes in Norway may in part explain why we do not observe large shifts in AMR. In such settings, including genotype in AMR surveillance of MRSA could contribute to improved/early detection of small-scale events or trends in AMR.

The most frequent AMR profile for Norwegian MRSA cases was cefoxitin resistance alone, reflecting that many of the most frequent *spa*-types were predominantly resistant to cefoxitin. However, we observed that some common genotypes had co-resistance to 2–3 antibiotics, including erythromycin, clindamycin, tetracycline and fluoroquinolones. The number of co-resistant strains (2–4 antibiotics) was also shown to be increasing moderately over time, and as such may present an increasing challenge for treatment of MRSA infections in the future. Norwegian guidelines for MRSA infections recommend treatment to be determined by AST as well as national or local resistance rates, however, for uncomplicated skin and soft-tissue infections, trimethoprim/sulfamethoxazole is the first choice, followed by clindamycin and tetracycline.^21^ We observed some correspondence between resistance patterns of MRSA strains and usage of antimicrobials (other than beta-lactams) in human medicine in Norway in the same period, especially for the most highly used antibiotic groups, tetracyclines and the MLS group (macrolide, lincosamide and streptogramin).^22^ One exception was the group sulfonamides and trimethoprim, for which usage is relatively high (0.84 defined daily doses per 1000 inhabitants/day in 2017), while resistance rates were low at only 2% in the study period. The trimethoprim/sulfamethoxazole-resistance rate has also been reported to be low in the same time period in the USA and Japan (around 1%).^23^ A contributing factor may be that resistance development to this combination antibiotic requires multiple adaptations, and it is well documented that this may adversely affect the fitness of *S. aureus.*^23^ Nevertheless, there are some successful global multi-resistant MRSA clones (clonal complex 239), which are very often resistant against trimethoprim/sulfamethoxazole.^24^

Resistance against erythromycin was significantly associated with infection in this study. Six of the *spa*-types (t002, t008, t021, t044, t437 and t657) that contributed the most to erythromycin resistance, were significantly associated with infection in a previous Norwegian study.^4^ These *spa*-types did not appear to be associated with higher age groups, and included both HA- and CA-MRSA.^4^ A possible explanation is the extensive use of macrolides for treating Gram-positive bacterial infections globally.^25^

A weakness of the study is that we do not have AST data from the earliest years of the study period, due to the fact that testing was not performed by the Norwegian MRSA reference laboratory at that time. Another potential challenge is the highly variable trend seen for ICR, which is probably caused by differences in reporting practice as well as general underreporting. According to EUCAST ICR strains should, however, be reported as clindamycin resistant or with a warning about possible failure of treatment.^15^ Vancomycin susceptibility was tested using a gradient strip test, which is known to overestimate MIC-values compared to the gold standard method micro broth dilution.^26^ Vancomycin resistance is reported to be rare in MRSA,^27^ and our findings are thus in concordance with this.

Our study identified a significant association between HA-MRSA, particularly within older adults and nursing homes, and co-resistance to ciprofloxacin/norfloxacin. Resistance mechanisms to fluoroquinolones in *S. aureus* primarily involves accumulation of chromosomal mutations and efflux pumps.^28^ The prevalence of ciprofloxacin/norfloxacin-resistant MRSA strains has been associated with use of fluoroquinolone antibiotics,^29^ which facilitates the emergence and spread of resistant strains within healthcare environments.^30^ In Norway, restrictive use of this antibiotic is therefore recommended. Despite these guidelines, adherence to the recommended usage has been observed to be suboptimal, with up to 92% non-compliance reported in Norwegian hospitals and nursing homes.^31,32^ Similar reports of non-adherence have also been documented in long-term care settings and for female urinary tract infections in the USA.^33,34^ Our results thus indicate that the frequent use of fluoroquinolones in elderly and nursing home patients may be driving resistance development in MRSA strains.

Some resistance profiles are generally linked to specific *spa*-types and variation of susceptibility will thus vary according to the genotype epidemiology. For antibiotic co-resistance and associations to place of acquisition of the MRSA strain, major endemic clones associated with these countries are likely the main contributors. For instance, the *spa*-type t008, which is endemic in the USA, exhibits high resistance rates to erythromycin and ciprofloxacin/norfloxacin.^35,36^ Thus, this presents a likely explanation for the association of MRSA co-resistance to these antibiotics of North-American strains in our study. Another example is the MRSA clone t437, predominantly associated with Vietnam and Thailand,^4^ which has been identified as a significant contributor to resistance against tetracycline. Similarly, MRSA t044, associated with Somalia,^4^ has been linked to resistance against fusidic acid and tetracycline. These findings show significant associations between specific antibiotic resistance phenotypes and geographic regions, thus emphasizing the importance of understanding local epidemiological contexts in addressing the challenge of antimicrobial resistance.

In summary, our findings highlight a complex landscape of MRSA resistance in Norway, characterized by a diverse genotypic population and stable longitudinal trends in resistance rates. However, the prevalence of co-resistance to one or more antibiotics among Norwegian MRSA strains is high and increasing. This study thus underscores the importance of continuous surveillance and adherence to antimicrobial guidelines.

## Supplementary Material

dlaf094_Supplementary_Data

## Supplementary data

Figures S1 to S4 is available as Supplementary data at *JAC-AMR* Online.
