# Supplementary material for: Antimicrobial resistance trends of methicillin-resistant Staphylococcus aureus in Norway from 2008 to 2017
Source: JAC Antimicrob Resist. 2025 Jun 6;7(3):dlaf094. doi: 10.1093/jacamr/dlaf094 (PMC12141746; doi:10.1093/jacamr/dlaf094)
Supplement: dlaf094_Supplementary_Data [file dlaf094_supplementary_data.docx]

**Supplementary figures and tables**

Figure S1: Number of MRSA cases with AST result interpreted as susceptible (S), intermediate (I), or resistant (R) or not tested (NT) per year in the study period.

**
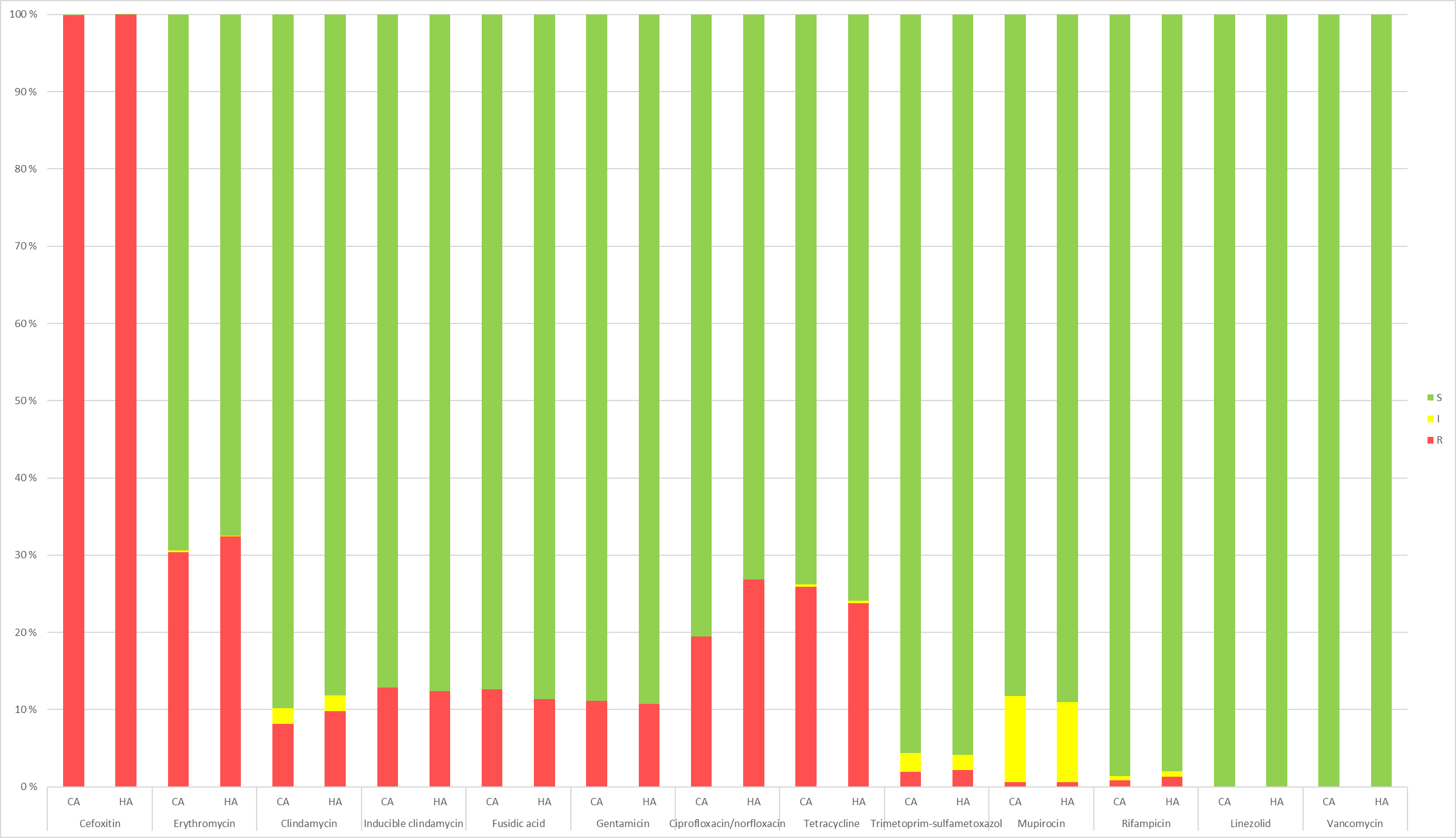
**

**Figure S2.** Proportion of HA and CA MRSA strains from Norway for the period 2009-2017 classified as susceptible (S), intermediate resistant (I) or resistant (R) to tested antibiotics.


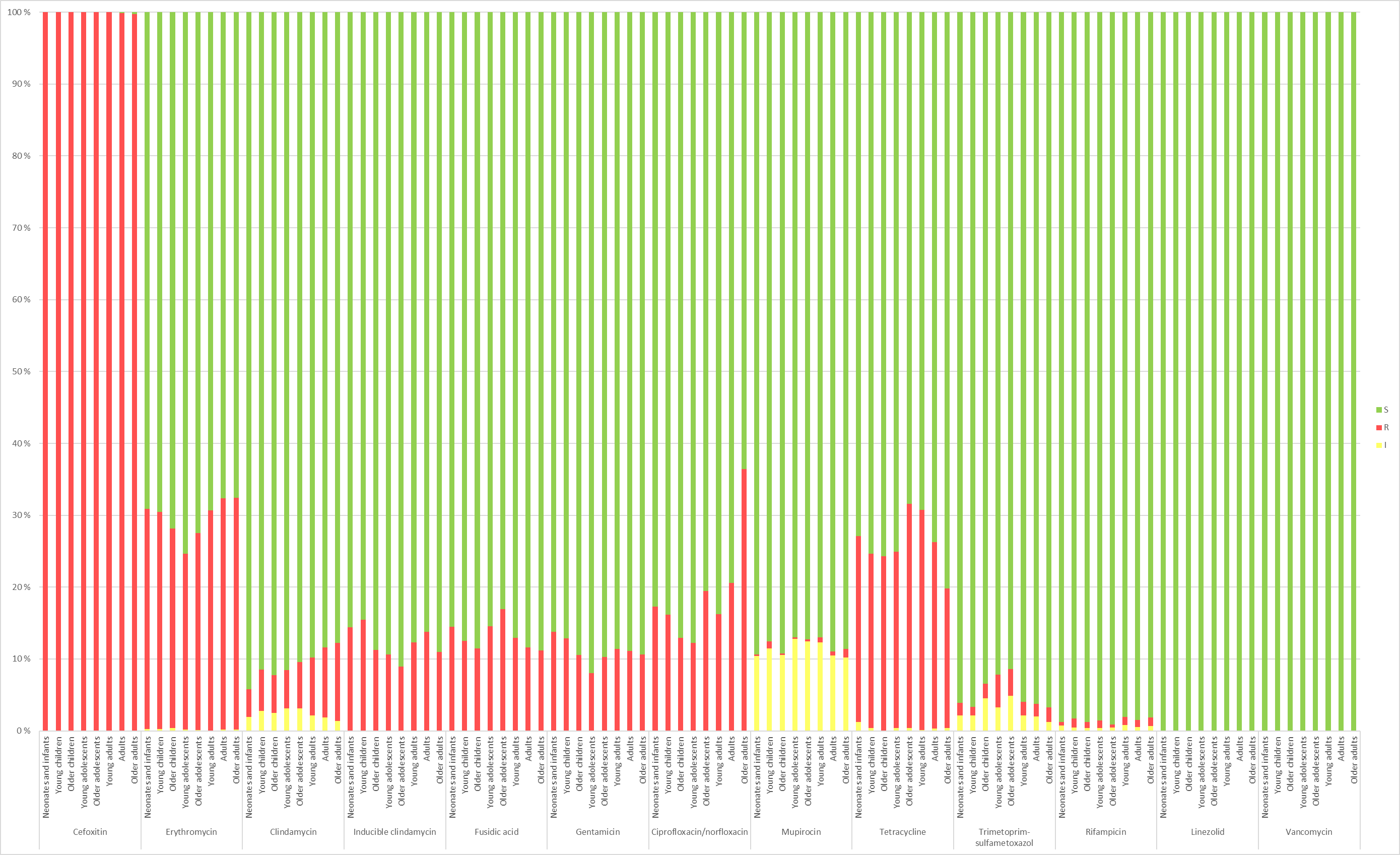


**Figure S3.** Association between age groups and antibiotic groups.


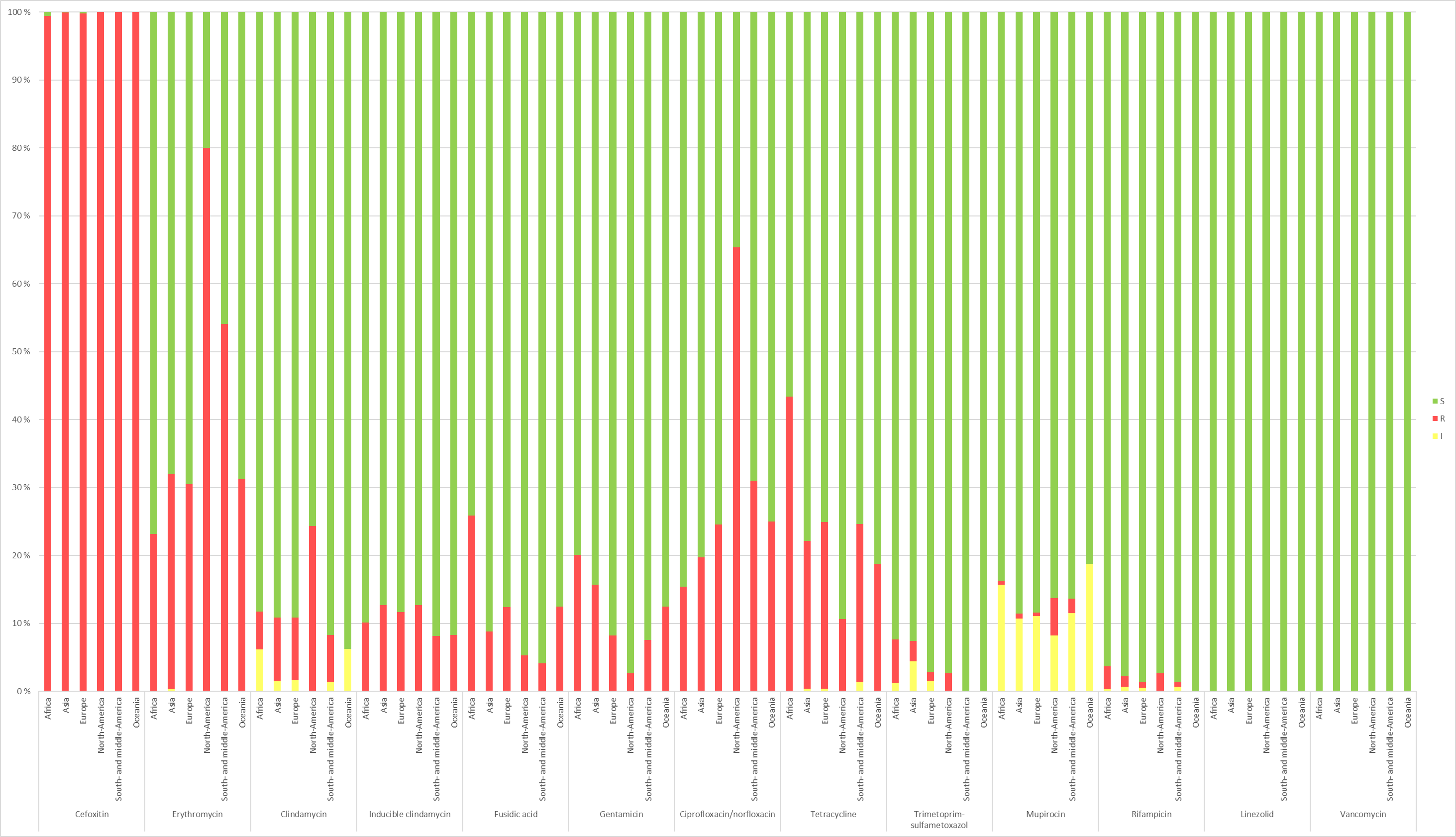


**Figure S4.** Association between continent and antibiotic groups.
